# Use of dried blood samples for monitoring hepatitis B virus infection

**DOI:** 10.1186/1743-422X-6-153

**Published:** 2009-09-29

**Authors:** Rosalia Lira, Angelica Maldonado-Rodriguez, Othon Rojas-Montes, Martha Ruiz-Tachiquin, Rocio Torres-Ibarra, Carlos Cano-Dominguez, Hilda Valdez-Salazar, Alejandro Gomez-Delgado, Onofre Muñoz, Ma-Teresa Alvarez-Muñoz

**Affiliations:** 1 Unidad de Investigacion Medica en Enfermedades Infecciosas y Parasitarias, Hospital de Pediatria, Centro Medico Nacional Siglo XXI, Instituto Mexicano del Seguro Social, Cuauhtemoc 330 Col. Doctores, Delegacion Cuauhtemoc, Mexico City, 06720, Mexico; 2 Clinica de Hepatitis, Hospital de Infectologia, Centro Medico Nacional La Raza, IMSS, Mexico

## Abstract

**Background:**

Hepatitis B virus (HBV) infection is a problem in several regions of the world with limited resources. Blood samples dried on filter paper (DBS) have been successfully used to diagnose and monitor several infectious diseases. In Mexico there is an urgent need for an affordable and easy sampling method for viral load (VL) testing and monitoring of chronic HBV infection. The purpose of this work was to validate the utility of DBS samples for monitoring HBV infection in patients from Mexico City.

**Methods:**

Matched samples of plasma and DBS on filter paper from 47 HBV infected patients from the Instituto Mexicano del Seguro Social (IMSS), were included. To evaluate the DNA stability and purity from DBS stored at different temperature conditions, samples from ten patients were stored at 4 degree, 25 degree, and 37 degree C for 7 days. After DBS elution and DNA extraction, the purity of these samples was determined measuring the O.D. rate 260/280. The DBS utility for molecular studies was assessed with PCR assays to amplify a 322 bp fragment from the "a" determinant region of the HBV "S" gene. The VL from all samples was determined to evaluate the correlation between plasma and DBS matched samples.

**Results:**

The quality of the DNA from DBS specimen is not adversely affected by storage at 4 degree, 25 degree and 37 degree C for up 7 days. Statistical ANOVA analyses did not show any significant difference. The same amplification efficiency was observed between DNA templates from samples stored at different temperatures. The Pearson correlation between the VL from DBS and plasma matched samples was 0.93 (p = 0.01). The SD was 1.48 for DBS vs.1.32 for Plasma, and an average of log_10 _copies/mL of 5.32 vs. 5.53. ANOVA analysis did not show any statistically significant difference between the analyzed groups (p = 0.92).

**Conclusion:**

The results provide strong evidence that the isolation and quantification of DNA-HBV from DBS is a viable alternative for patient monitoring, and molecular characterization of the virus variants circulating in Mexico.

## Background

Blood samples dried on filter paper have been successfully used to diagnose and monitor several infectious diseases. The dried blood spots (DBS) have been used to detect antibodies, and to purify nucleic acids and other molecules. Filter papers were initially used for the screening of newborn metabolic disorders [1]. Currently, they have proved useful in detection, quantification and identification of a variety of infectious pathogens, including viruses (HIV and CMV) [2-4], and different parasitic infections [5]. DBS samples are a simple and inexpensive sampling method, especially useful for blood collection in resource-poor settings with limited access to diagnostic facilities. The main advantage of DBS samples over routine blood samples is that only a small quantity of blood, typically 50 μl, is required to make one dried blood spot. They are easy to obtain, stable for long periods of time, and can be transported to a reference laboratory at minimal cost [6-8].

Hepatitis B virus (HBV) infection is a problem in several regions of the world with limited resources. The diagnosis and monitoring of HBV infection is generally based on the determination of serologic markers, and viral load quantification; however, molecular characteristics such as genotype and genetic variants are not used routinely.

Based on the complete genome sequences, HBV has been classified into eight genotypes, A to H [9,10]. In Mexico, where the incidence of disease is increasing [11], genotype H is predominant [12,13]. Since it is the most recently described genotype, information about the genetic characteristics and molecular variants circulating in our country is limited.

In spite of the urgent need for an affordable and easy sampling method for viral load testing and monitoring of chronic HBV infection, there is only one report on the role of DBS in evaluation of patients infected with HBV. In this study, the viral load was 1 log lower than those detected in serum samples. However, DNA amplified from these samples proved to be useful in the identification of specific mutations in the precore and polymerase motifs [14]. We were interested in validating the use of DBS samples as an alternative to plasma for monitoring HBV infection and the potential utility for molecular studies. Our data support the utility of the DBS sampling for monitoring HBV infection. Therefore we strongly recommend this method of specimen collection for HBV infection monitoring in low resources countries.

## Materials and methods

### Patients

This study included 47 hepatitis B surface antigen -positive (HBsAg) patients from the Instituto Mexicano del Seguro Social (IMSS). Local ethical and scientific committees of the Institute approved the procedures and the protocol. Blood samples for the study and basic demographic data were obtained under informed consent from each subject (Table 1).

**Table 1:** Clinical and virological patient data

**Patients**	**n = 47**
Sex M: F	36:11
Age	35.7 ± 12.9
Plasma viral load	1,280 - 68,000,000
(copies/mL)	log_10 _3.1- 7.8
HBeAg+	38 (72.2%)
Anti-HBeAg	15 (27.8%)
Anti-HBc	49 (90.7%)

### Plasma and DBS samples

Two tubes of EDTA-anticoagulated whole blood and one tube without anticoagulant were collected by venipuncture from each subject. Plasma aliquots were obtained by centrifugation of the 8 mL whole blood at 4000 rpm for 20 min. The supernatant was stored at -70 degree C until use. Replicate sets of DBS samples were prepared dropping 50 μl of whole blood in each circle (5 spots per card) of the filter paper (SS&S903, Schleicher & Schull). They were air-dried for 4 h at room temperature and then placed into zip-locked bag along with silica gel desiccant sachet, and stored at -20 degree C until processing.

### HBV-DNA DBS stability storage at different temperatures

Ten different patient samples were kept at 4, 25 and 37 degree C for 7 days, before storing at -20 degree C until use. One set of samples was stored since the beginning at -20 degree C for comparison.

### Nucleic acid isolation from dried blood samples and plasma, and PCR assay

In order to determine the quality and utility of the DNA extracted from DBS samples stored at different temperatures, two approaches were used. The DNA quality was measured by spectrophotometer and the utility of the sample for molecular analyses was evaluated by PCR amplification of a fragment from the determinant "a" region of the genome [13,15].

#### a) Nucleic acid isolation

For plasma samples, extraction was performed using the QIAamp^® ^Ultrasens^® ^Virus kit (QIAGEN GMBH, Germany), with 100 μl of plasma following the manufacturer's instructions. Scissors were used to cut one or two spots for each sample (50 μl/spot), and the blood was eluted and the DNA extracted using the QIAamp^® ^DNA micro kit (QIAGEN GMBH, Germany), following the manufacturer's instructions. The quality of the extracted DNA was assessed by spectrophotometry (NanoDrop^® ^ND-1000 Spectrophotometer v3.0.1, USA.) and the optical density (O.D.) 260/280, was calculated.

#### b) Amplification by PCR

In order to determine the utility of the extracted nucleic acids from DBS samples for molecular studies, the DNA extracted was utilized as a template for PCR to amplify a fragment from the determinant "a" region of the HBV "S" gene using primers and conditions previously reported [15]. Sequences of the outer primers and their relative positions were as follows: HBV1-sense, 5'-CGC TGG ATG TGT CTG CGG CGT-3', position 371-391, and HBV2-antisense, 5'-CGA ACC ACT GAA CAA ATG GCA CT-3', position 682-704. Briefly, 10 μL of DNA extracted from plasma and DBS samples were mixed with 40 μL of master mix containing 1 × PCR buffer, 50 pmoles of each primer, 0.2 mM dNTPs, 2.5 mM MgCl_2_, and 2.5U Taq polymerase (Amplificasa^® ^BIOGENICA, Mexico). PCR amplification was performed using HBV1-sense and HBV2- antisense outer primers as described previously [13]. A 322 bp product was visualized by ethidium bromide staining on a 1% agarose gel.

#### c) Plasma viral load (VL) quantification

The levels of HBV DNA in 100 μl of plasma were quantified by using the Amplicor HBV Monitor kit (Roche Molecular Systems, Inc.) according to the manufacturer's instructions.

### DBS viral load (VL) quantification

The blood was eluted from a single filter spot for each sample (50 μl). For plasma VL below log 10^4^, two discs were used. The eluted material was processed using the QIAamp^® ^DNA micro kit (QIAGEN GMBH, Germany) following the manufacturer's instructions. The final volume of extracted DNA elution was 50 μL in dH_2_O HBV Monitor Cobas Amplicor v 1.5 (Roche Diagnostics, New Jersey, EU). Samples with plasma VL > 10^6 ^were diluted 1:100 with saline solution. A 1:1000 dilution was used in samples with VL > 10^7^.

### Statistical analyses

The statistical analyses were performed using SPSS 10.0 version software (SPSS Chicago IL). Pearson correlation was used to determine the association between VL from DBS and plasma. The ANOVA analysis was used to evaluate differences between the VL of groups stored at different temperatures. Kruskal-Wallis Test was used to evaluate differences between the O.D. measurements for the DBS extracted DNA quality.

## Results

### DBS genomic DNA integrity and storage temperature stability studies

To evaluate the utility of the HBV DNA extracted from DBS to monitor viral load (VL) and perform molecular analyses, three assays were done with ten different samples stored for 7 days at 4 degree, 25 degree and 37 degree C, and compared with samples stored at -20 degree C.

#### a. DNA quality measurements

The quality of the DNA extracted from the 10 different samples stored at 4 different temperatures was obtained by duplicates of O.D. 260/280 measurements. The ANOVA test (p 0.67) did not show significant difference between samples. The quality of the DNA extracted from DBS is not adversely affected by storage at 4 degree C, RT, and 37 degree C for up 7 days.

#### b. PCR amplification

**Figure 1:**
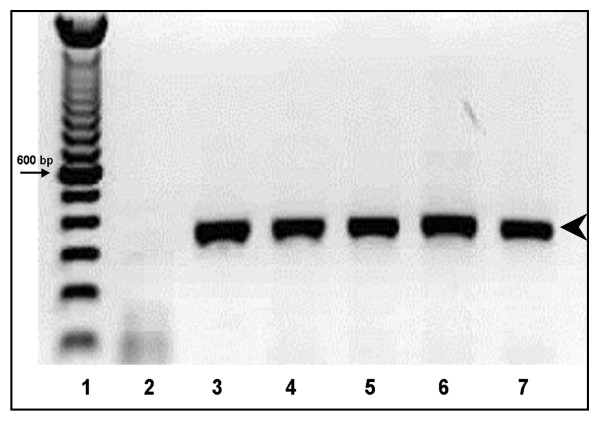
**Stability of the dried blood samples**. PCR amplifications of HBV DNA from DBS. 1% Agarose gel showing a 322 bp fragment of the HBV-"a determinant" amplified from DBS samples stored at different temperatures. 1. DNA molecular markers; 2 negative control; 3 Plasma at -70 degree C; 4 DBS at -20 degree C; 5, DBS at 4 degree C; 6, DBS at 25 degree C; 7, DBS at 37 degree C. The arrowhead on the left denotes the 322-bp amplified product.

To evaluate the efficiency of amplification a PCR product of 322 bp from the HBV determinant "a" genome was amplified. Concordance between DBS stored at different temperatures and plasma samples for HBV-PCR amplification was optimal, because all the samples were successfully amplified (Fig 1).

#### c. Correlation between HBV-DNA VL in DBS samples stored at different temperatures and frozen plasma

**Figure 2:**
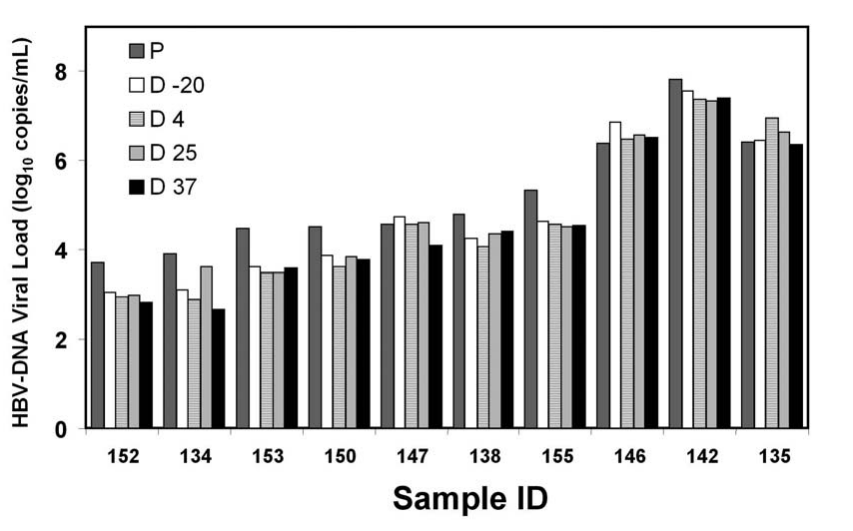
**Viral Load correlation between DBS and plasma matched samples at different storage temperatures**. The VL was assessed by Cobas Monitor Amplicor, and the values were transformed to Log_10_. VL values for plasma samples from ten different patients (sample ID) stored at -70 degree C (P), and paired blood spots samples stored at 4 degree C (D 4), 25 degree C (D 25), 37 degree C (D 37), and -20 degree C (D-20) for up to 7 days, and removed to -20 degree C until analysis.

First, the dried whole blood spot stability was evaluated measuring the VL from samples stored at 4 degree, 25 degree, and 37 degree C. The data (Fig 2) showed no difference in VL values from matched samples stored at different temperatures compared to the gold standard in plasma; the 5.32 and 5.53 log differences were not statistically significant by the ANOVA test. The CI 95% for the gold standard in plasma was (3.1-7.83), for DBS at: -20 degree C (3.05-7.57); at 4 degree C (2.91-7.39); at 25 degree C (2.97-7.41) and at 37 degree C (2.67-7.83). The DBS storage temperature at 4 degree, 25 degree and 37 degree C for up 7 days did not affect the VL measurements.

### Correlation between viral load (VL) of plasma and dried blood samples

**Figure 3:**
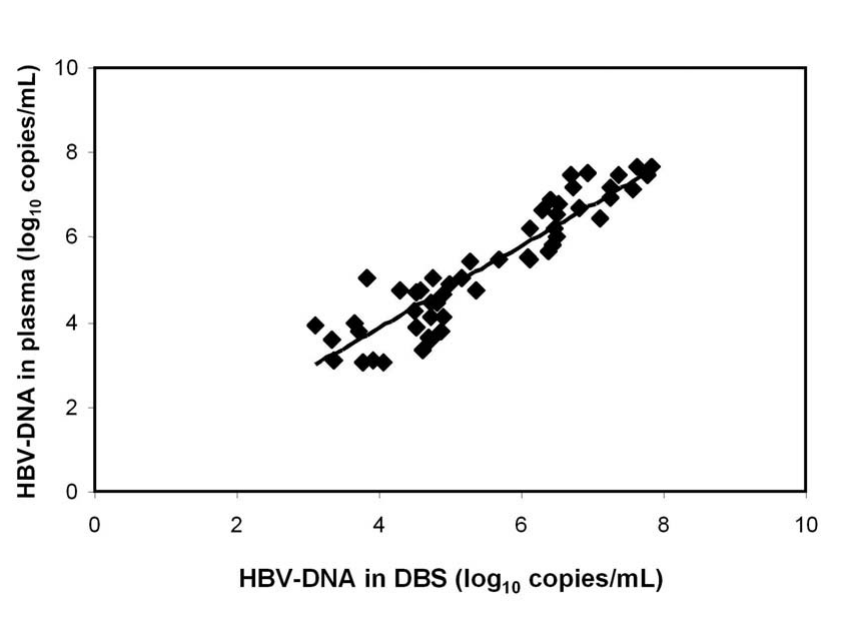
**Log-log_10 _graph showing the linear regression comparing VL data obtained from 47 matched samples of DBS vs. Plasma**. Log_10 _measurements of the HBV DNA in DBS and Plasma samples, assessed by Cobas Monitor Amplicor. The values for the DBS are plotted against the values for the matched plasma samples. The linear correlation between the two samples is shown (R^2 ^= 0.86). The Pearson correlation coefficient was 0.93.

To validate the utility of DBS, VL determination of matched samples from plasma and DBS was performed. The average value for VL in plasma was log_10 _5.48 with a SD of 1.32, and in DBS was 5.29 with SD of 1.46. The sensitivity of HBV DNA detection in DBS was the same as in plasma samples (100%). In evaluating the VL only one disc per sample (50 μl) was used, and plasma value was obtained from 100 μl, therefore a normalization factor of 2 was used for the final DBS values. In addition, it was important to use a 1:100 dilution when samples with VL concentration in plasma was log_10 _6.1-6.9, because samples without dilution did not correlate with paired plasma sample values. In samples with log_10 _from 6.9 to 7.8, a 1:1000 dilution was used. These modifications allowed us to obtain a good linear correlation in a log-log plot between HBV-DNA concentrations in DBS versus plasma samples (Fig. 3). We also found a high Pearson correlation coefficient (0.93). This result clearly indicated that DNA HBV concentration from DBS is highly comparable to plasma paired values.

## Discussion

Traditionally, the monitoring of HBV infection is done by serological assays involving serum and plasma samples that require frozen storage conditions. Blood samples must be processed within 6 hours of collection. If the assay is not available immediately, the samples must be frozen at -20 for serum, or -70 degree C for plasma. In developing countries where cold storage and transportation present special problems, the use of DBS should be considered. The introduction of whole blood samples in filter paper or DBS in clinical samples has improved the monitoring and sentinel surveillance of various infectious diseases. Several studies have been done to demonstrate the efficiency of the method for collection and long term storage for field samples [3,16-19]. The use of DBS for monitoring of HIV-1 infection has shown that this sampling method is useful not only for a safe and easy manipulation of a contagious sample, but also for diagnosis and epidemiologic monitoring of the infection [2,14,20].

In the case of HBV infection, DBS samples have been used for detection of viral antigens and antibodies [21,22]. Jardi et al, demonstrated the application of DBS samples for HVB DNA quantification and genetic variant analysis [14]. They found that sensitivity of HBV DNA quantification in DBS samples was 1 log lower than in serum samples. The detection limit of the DBS assay was 2 × 10^3 ^HBV-DNA copies/mL, and only A, D and F genotypes were assayed. Interestingly, by using the plasma concentration as a reference value to determine if the sample should be diluted, our results showed a very good correlation between VL of plasma vs. DBS samples. According to the manufacturer Cobas Amplicor HBV Monitor Roche, samples with high viral loads, e.g. higher than log_10 _5.1, will saturate the amplification system and must be diluted in order to obtain a correct value. It is recommended to dilute samples from 1:10, 1:100 and 1:1000 depending on the result. By using this method, the sensitivity of was the same for plasma and DBS samples, supporting the use of these samples for VL determinations and monitoring infection in countries with low resources.

In Mexico, DBS sampling method is not used for monitoring HBV infection, even though the incidence of this disease is increasing [11]. We think it is urgent to introduce a simple and non-costly sampling method in order to improve the molecular diagnosis and monitoring of HBV infection and reduce the cost in the management of the disease. We also found that different storage temperatures up to 7 days did not affect the quality of the HBV DNA. The use of these samples is readily applicable in countries like Mexico, where the cold storage and transportation is expensive or sometimes unavailable. It is very important to establish a Reference Center where the samples from all over the country could be collected and assayed for monitoring infection and perform genetic analyses. Remarkably, samples stored at room temperature (25 degree C) are an excellent option to perform viral load quantification and other molecular studies, like PCR amplification to detect genomic differences in isolates from different regions. Several investigators have reported the DBS storage at different conditions did not affect significantly the VL determinations [6-8]. The time of storage is also an advantage in the use of these samples, because it has been reported that RNA HIV-1 stored up 28 years did not affect the VL determinations. It is clear that extrapolations to the use of DBS are a valuable option.

## Conclusion

These results provide strong evidence that the isolation and quantification of HBV from samples collected on filter paper is a viable alternative to the routine freezing method for the transportation of clinical plasma or serum samples, and to perform monitoring of the virus variants circulating in Mexico.

## List of abbreviations

HBV: hepatitis B virus; DBS: dried blood spots; HBsAg: Hepatitis B surface antigen; HBeAg: Hepatitis B e antigen; HBcAg: Hepatitis B core antigen; VL: viral load; IMSS: Instituto Mexicano del Seguro Social.

## Competing interests

The authors declare that they have no competing interests.

## Authors' contributions

**RL **participated in the study design and participated in drafting and discussing the manuscript. **AMR, ORM, and HVS **performed the experiments and discussing the manuscript. **MRT and OM **participated in the study design and discussing the results. **RT and CC **provided patients and all participated in drafting and discussing the manuscript. **AG **performed the statistic analysis. **MTAM **participated in the study design, and in drafting and discussing the manuscript. All authors have read and approved the final manuscript.

## Authors' information

RL. Unidad de Investigacion Medica en Enfermedades Infecciosas y Parasitarias. Hospital de Pediatria, Centro Medico Nacional Siglo XXI, Instituto Mexicano del Seguro Social, Cuauhtemoc 330 Col. Doctores, Delegacion Cuauhtemoc, Mexico City, MEXICO 06720.
